# Comparative Analysis of Triticeae Satellite Repeats Using Low-Coverage Sequencing, qPCR, and FISH

**DOI:** 10.3390/ijms27167362

**Published:** 2026-08-18

**Authors:** Anna I. Yurkina, Pavel Yu. Kroupin, Daniil S. Ulyanov, Viktoria M. Sokolova, Gennady I. Karlov, Mikhail G. Divashuk

**Affiliations:** All-Russia Research Institute of Agricultural Biotechnology, Timiryazevskaya St., 42, 127434 Moscow, Russia; aaaaaa3197@gmail.com (A.I.Y.); uldas1508@gmail.com (D.S.U.); vika-kuz367@yandex.ru (V.M.S.); karlov@iab.ac.ru (G.I.K.); divashuk@gmail.com (M.G.D.)

**Keywords:** satellite DNA, repeatome, *Elymus*, *Dasypyrum villosum*, quantitative PCR, fluorescence in situ hybridization (FISH), wheat introgression lines

## Abstract

Satellite DNA is a dynamic component of plant genomes and a valuable source of cytogenetic markers, but its diversity and chromosomal distribution in polyploid Triticeae remain insufficiently studied. Here, low-coverage whole-genome sequencing, graph-based repeat clustering, quantitative PCR, multivariate statistics and fluorescence in situ hybridization (FISH) were used to identify and characterize satellite repeats in *Elymus* and related Triticeae species. Sixteen repeat clusters (E1–E16), with monomer lengths of 118–667 bp, showed distinct taxonomic distributions and copy-number profiles across 14 species. Correlation analysis, principal component analysis and hierarchical clustering revealed concerted variation among repeats and separated the perennial taxa *Elymus* and *Pseudoroegneria* from *Triticum*, *Secale*, *Hordeum* and *Dasypyrum*. Spearman correlation analysis identified E7 and E9 as putative candidates associated with St/StY genomic backgrounds, whereas E10 was identified as a putative candidate associated with the H genome. These statistical associations require independent cytogenetic validation. Contrasting copy numbers of E6 and E11 in bread wheat cv. Chinese Spring versus *Dasypyrum villosum* (L.) Candargy identified them as V-genome candidates. FISH localized E6 to the terminal regions of chromosomes 3VL, 4VS and 7VS, and E11 to 4VL. Karyotyping further revealed that two lines previously considered as wheat-*D. villosum* addition lines were in fact substitution lines: W3 was identified as a 3V(3D) substitution line and W4 as a 4V(4B) substitution line, whereas W7 retained its 7V addition status. These results expand the set of chromosomal markers for comparative genomics and introgression analysis in wheat.

## 1. Introduction

The genomes of representatives of the tribe Triticeae are among the largest and most complexly organized of all flowering plants. A substantial proportion of their nuclear DNA consists of repetitive sequences, collectively referred to as the repeatomes [1]. This repeatome comprises mobile elements (transposons and retrotransposons), clusters of ribosomal RNA genes, and satellite repeats—tandemly organized non-coding sequences located predominantly in heterochromatic chromosomal regions such as centromeres, pericentromeres and subtelomeric areas [2]. Although these sequences were long regarded as functionally inert ‘junk’ DNA, molecular-genetic and cytogenomic studies of recent years have convincingly demonstrated their involvement in maintaining the structural and functional organization of the genome. Satellite DNA has been shown to participate in centromere and kinetochore formation, the spatial organization of chromatin, the regulation of gene transcriptional activity, and processes of interchromosomal interaction [3,4,5]. Moreover, the rapid evolutionary rate of satellite repeats makes them among the most sensitive indicators of genome divergence and speciation processes. According to the satellite DNA library hypothesis, closely related species inherit a common set of satellite families, but changes in their copy number and chromosomal localization occur during evolution, leading to the formation of species-specific repeat profiles [6,7].

Advances in high-throughput sequencing and specialized bioinformatic approaches have made it possible to perform comprehensive repeatome analyses even from low-coverage sequencing data [8,9]. Substantial progress in the study of plant repetitive sequences has been achieved through the development of graph-based read-clustering methods implemented in the RepeatExplorer and Tandem Repeat Analyzer (TAREAN) software packages [8,10]. These tools allow the identification of major repeat families without the need for complete genome assembly. The application of such approaches has considerably broadened our understanding of satellite DNA diversity in Triticeae and other grasses [7,11].

The evolution of repetitive sequences under polyploidization is of particular interest, as polyploidization is one of the key mechanisms driving diversification within the tribe Triticeae. Following the union of different subgenomes in allopolyploids, extensive repeatome rearrangements occur, including the amplification of some repeat families and the elimination of others [12,13,14,15,16]. These processes can lead to rapid changes in heterochromatin structure and to the formation of new chromosomal patterns of satellite DNA distribution [17]. Studies conducted on wheat and its wild relatives have shown that individual satellite repeat families display subgenome specificity and can be used to identify the genomic components of polyploid species [9,18].

Real-time quantitative PCR (qPCR) is a method for estimating the copy number of repetitive sequences in the genome that has gradually replaced more labor-intensive classical approaches (Southern blot, dot-blot) [18]. In addition to requiring less labor and time, qPCR offers higher accuracy, since it allows work under conditions of strict primer specificity that exclude non-specific hybridization. The method enables simultaneous assessment of the copy number of several repeat families across a series of samples, including species with different ploidy levels and genome formulas [19,20,21,22].

The application of qPCR opens broad possibilities for studying repeatomes and addressing problems of comparative and evolutionary genomics in grasses [9]. Quantitative assessment of satellite repeat copy number allows, first, the differentiation of species-specific and conserved families; second, the detection of patterns of their amplification and elimination during genome divergence; and third, the use of individual repeats as molecular markers. This approach becomes especially valuable in studies of polyploidization: comparing repeat copy numbers between diploid donors and allopolyploid species makes it possible to reconstruct post-polyploidization repeatome rearrangements. The effectiveness of the method has been confirmed in *Aegilops* and *Thinopyrum* species, where its application helped clarify subgenome origins and trace evolutionary transformations of chromatin [9].

Another important application of qPCR is the identification of subgenomes in species of disputed origin. A high copy number of a particular repeat in a putative genome donor and related polyploids allows it to be regarded as a marker of subgenomic affiliation. Such an approach was used in studies of species of the genus *Dasypyrum*, where analysis of the copy number of the pHv-961 repeat helped clarify the genomic composition of *Dasypyrum breviaristatum* (H. Lindb.) Fred [19].

In addition, qPCR represents an effective tool for the preliminary screening of candidates for FISH analysis [9,23]. Assessment of repeat copy number allows the selection of the most promising sequences capable of forming distinct hybridization signals, which substantially reduces the volume of labor-intensive cytogenetic experiments. Thus, current research indicates that satellite DNA is not a passive genomic component but a dynamic and functionally significant part of the plant repeatome. The high variability of these sequences, their close association with chromosome architecture, and their sensitivity to evolutionary processes make satellite repeats a valuable tool for comparative genomics, phylogenetics, and studies of the origin of polyploid species within the tribe Triticeae.

Despite considerable progress in the study of the Triticeae repeatome, several questions remain open. In particular, comparative analysis of satellite DNA in species of the genera *Elymus*, which possess a complex polyploid nature and an ambiguous genomic formula, has so far been conducted only to a limited extent [24].

The aim of the present study was to characterize the diversity and copy number of satellite repeats in *Elymus* and related Triticeae representatives and to evaluate the prospects of the identified repeats as subgenome- and chromosome-specific cytogenetic markers.

## 2. Results

### 2.1. Characterization of the Identified Satellite Repeats

In total, 16 repeat clusters (E1–E16) were identified using two complementary repeatome-analysis approaches. Clusters E1–E7 were obtained from the analysis of the six taxa constituting the main sequencing dataset (*Elymus tschimganicus* (Drobow) Tzvelev, *E. tsukushiensis* Honda, *E. dahuricus* Turcz., *E. caninus* (L.) L., and the Afghan and Russian accessions of *E. repens* (L.) Gould), whereas clusters E8–E16 were identified through three comparative assemblies designed to reveal repeats associated with particular genomic components. Specifically, E8 and E14 were identified by comparing *E. repens^(Afg)^* (StStH) and *E. tsukushiensis* (StHY), E9–E13 by comparing *E. repens^(Rus)^* (StStH) and *E. tsukushiensis* (StHY), and E15–E16 by comparative analysis of *Elymus barbicallus* (Ohwi) S.L.Chen, *Elymus pendulinus* (Nevski) Tzvelev (StY), and *Elymus arizonicus* (Scribn. & J.G.Sm.) Gould (StH). The identified repeats differed in monomer length, taxonomic distribution, and sequence similarity to previously characterized Triticeae repeats. Detailed BLAST results, including sequence accession numbers, percentage identity, query coverage, and E-values, are provided in Tables S1–S4; therefore, only the most informative and biologically relevant homologues are highlighted below.

Clusters E1, E2, E6 and E7 were detected in all six taxa, whereas E4 was absent only from *E. dahuricus.* In contrast, E3 and E5 showed a more restricted distribution. Monomer lengths ranged from 118 nt for E2 to 667 nt for the longest E1 variant. Overall, E1–E7 showed similarity to previously characterized satellite and other repetitive sequences from several Triticeae genera, indicating different degrees of conservation across the tribe (Table S1). Particularly high similarity was observed for E6, which showed 98.31% identity to CL69 of *Pseudoroegneria spicata* (Pursh) Á. Löve and 96.25% identity to the previously characterized *Dasypyrum* repeat CL169 [25]. E7 was most similar to the *Pseudoroegneria tauri* (Boiss. & Balansa) Á.Löve satellite CL185 (97.86%).

Comparative repeatome analysis of *E. repens^(Afg)^* (StStH) and *E. tsukushiensis* (StHY) identified clusters E8 and E14 (Table S2). E8, with a monomer length of 376–380 nt, was moderately enriched in *E. tsukushiensis* (57.81% of reads), whereas E14 (554–571 nt) showed a pronounced predominance in *E. repens^(Afg)^* (86.95%). Both repeats were detected across the six taxa studied. Representative homologues included the *Aegilops crassa* Boiss. satellite CL244 for E8 (98.79% identity) and the *Elymus libanoticus* (*P. libanotica*) (Hack.) D.R.Dewey satellite StLIB96 for E14 (93.92%).

Comparative analysis of *E. repens^(Rus)^* (StStH) and *E. tsukushiensis* (StHY) identified clusters E9–E13 (Table S3). E9 and E11 were strongly enriched in *E. tsukushiensis* (93.02% and 97.85% of reads, respectively), whereas E12 and E13 predominated in *E. repens^(Rus)^* (65.90% and 66.97%, respectively); E10 was represented at nearly equal proportions in the two species. The most informative homology relationships included E10 with the *Aegilops tauschii* Coss. microsatellite P523 (92.07%), E11 with StY_90_c sequences of *Elymus ciliaris* (Trin.) Tzvelev and *Elymus grandis* (Keng) S.L.Chen (89.30–98.61%), and E12 with *Hordeum vulgare* L. repetitive sequences. In addition, E11 showed 74.27% identity to the previously characterized *Dasypyrum* repeat CL135 [25]. E13, similar to E14, showed the highest similarity to StLIB96 of *E. libanoticus* (93.92%).

Finally, comparative repeatome analysis of *E. barbicallus*, *E. pendulinus* (StY) and *E. arizonicus* (StH) identified clusters E15 and E16, both of which were absent from the six taxa of the main dataset (Table S4). E15 was represented at similar proportions in the three species (26.19–34.23%) and showed high similarity to several transposon and retrotransposon sequences, suggesting a possible mobile-element-related origin. E16 was most abundant in *E. arizonicus* (41.67%) and had no annotated homologues in the NCBI database, indicating that it may represent a previously undescribed repetitive sequence.

### 2.2. Assessment of Repeat Cluster Copy Number

**Figure 1:**
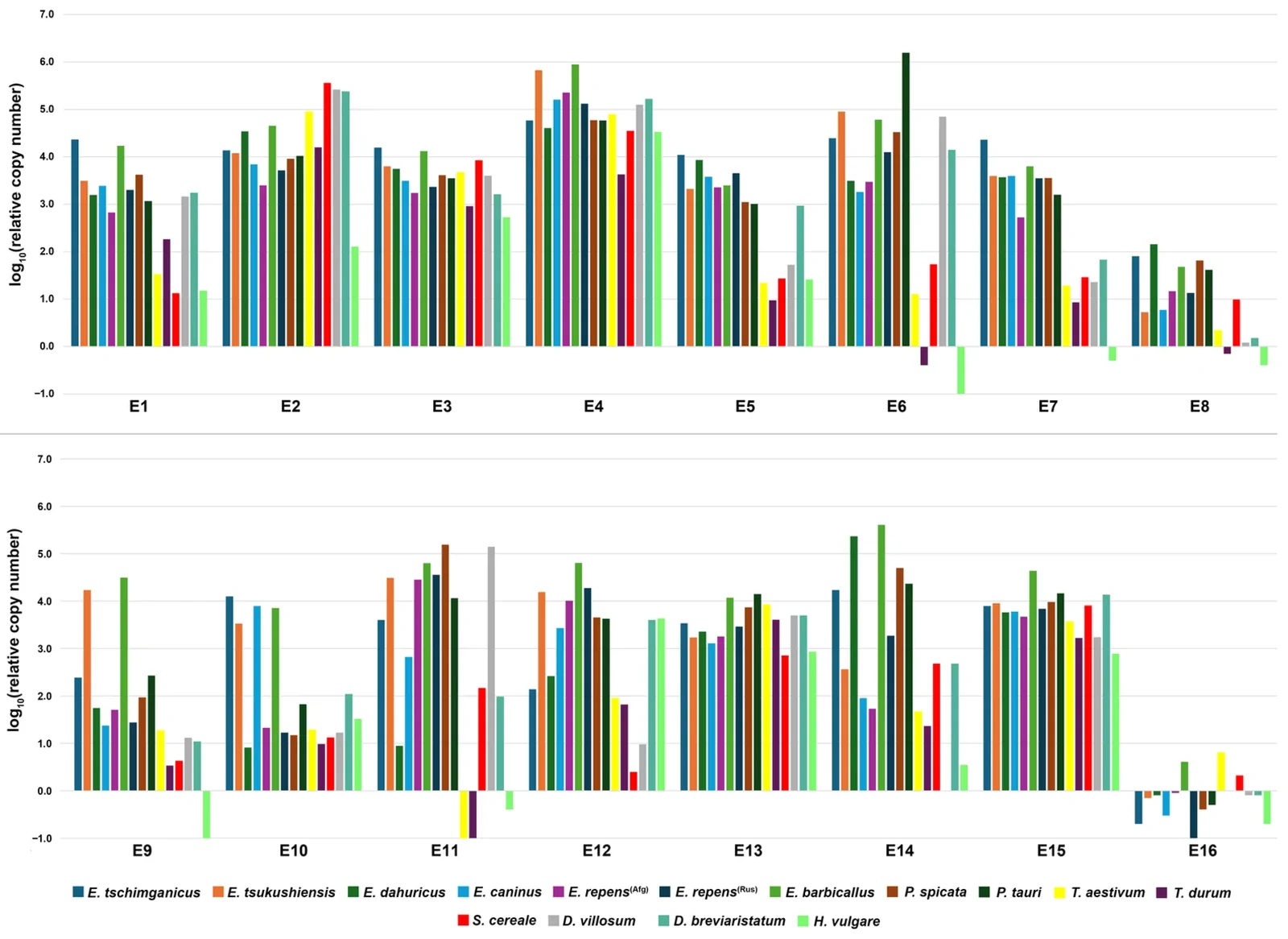
Relative copy number of satellite repeats in the studied species, expressed as the decimal logarithm.

The relative copy-number data for the 16 satellite repeats across 14 species, calculated relative to the single-copy reference gene *VRN1*, are presented in Tables S5 and S6. For ease of comparison, the results were expressed as the decimal logarithm of relative copy number (Figure 1).

Hereafter, «copy number» refers to the decimal logarithm of relative copy number. As before, repeats were conventionally divided into groups with low (≤2), medium (>2 and <4) and high (≥4) copy number. The level of variability was assessed by the coefficient of variation: low—below 0.1, medium—0.10–0.25, and high—above 0.25.

The most stable repeats were E3, E4, E13 and E15, whose coefficient of variation was 0.1 or less. Among these, repeat E4 was characterized by the highest copy number in most species (mean value 5.3), whereas E3, E13 and E15 predominantly showed medium copy-number levels (3.7–4) with minimal interspecific variability.

A medium level of variability was characteristic of repeat E2 (0.18). This repeat was distinguished by a high mean copy number (4.9); in most species the values exceeded 4, whereas in some species a decrease in copy number was observed: the maximum copy number was observed in *Secale cereale* L. (5.6), and the minimum in *H. vulgare* (2.1).

High variability was characteristic of repeats E1, E5, E6, E7, E8, E9, E10, E11, E12 and E14, whose coefficients of variation ranged from 0.28 to 0.55. Among these, the highest mean copy numbers were found for E6 (5.1), E11 (4.5) and E14 (4.7), although their values differed among the species studied. Repeat E8 was distinguished by the lowest mean copy number (1.5), ranging from very low to moderate values, which resulted in the maximum variability among most of the studied repeats (0.55). Repeats E1, E5, E7, E9, E10 and E12 on average showed medium copy numbers (3.3–3.9), but displayed pronounced interspecific differences.

E16 stands out for its abnormally high variability, resulting from the fact that in many species its copy number is close to zero or negative (logarithm of relative content below 0), while the maximum value does not exceed 0.8 (*Triticum aestivum* L.).

#### 2.2.1. Correlation Structure of Satellite Repeat Copy-Number Patterns and Their Associations with Genomic Groups

**Figure 2:**
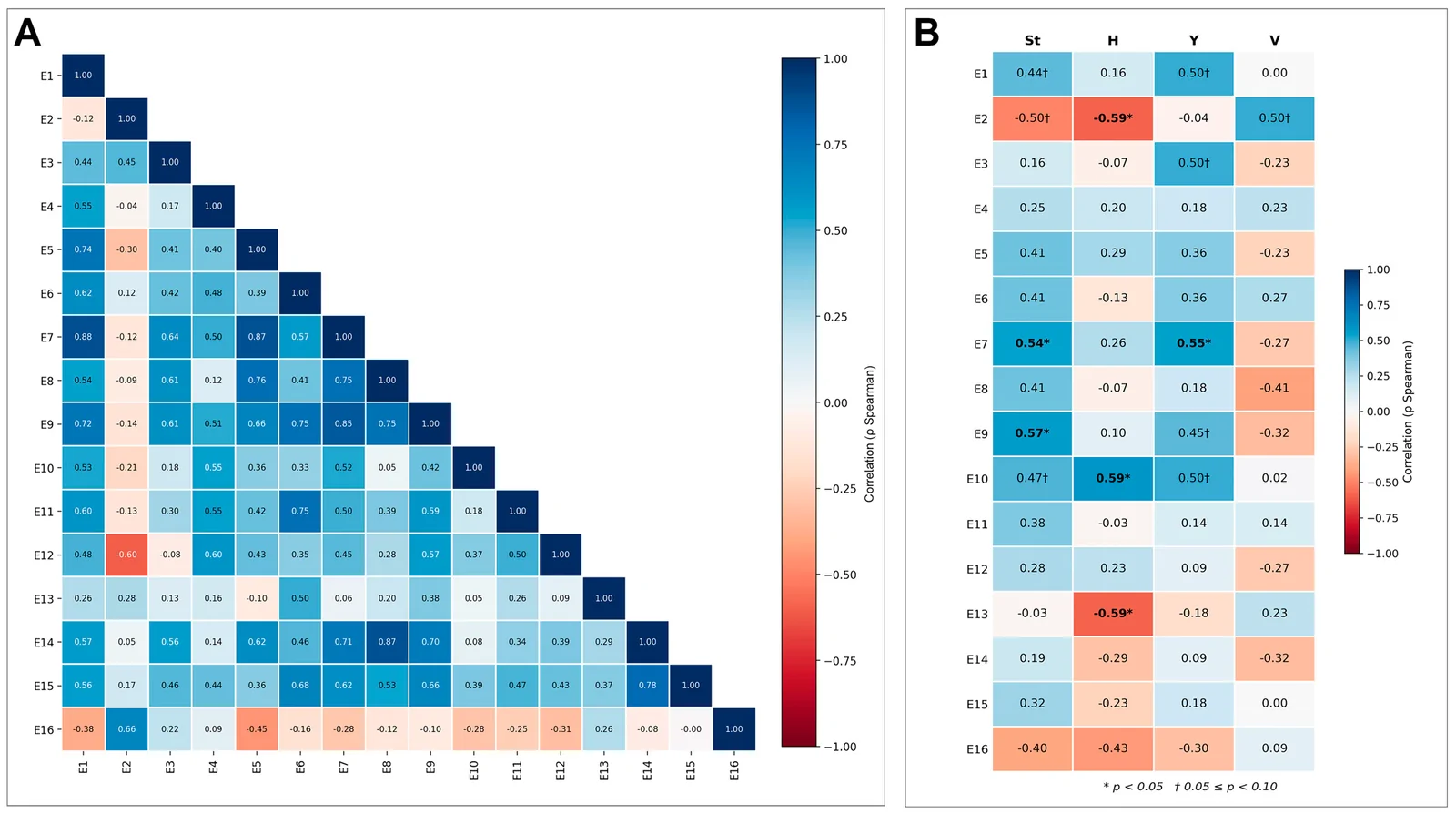
Correlation structure of copy-number patterns of the 16 satellite repeats (E1–E16) in the studied species: (**A**) Correlogram of the Spearman rank correlation coefficient matrix among repeats. The color scale reflects the strength and direction of the association (from dark blue, ρ > 0.8, to dark red, ρ < −0.6); numerical ρ values are indicated in the cells, with values significant at *p* < 0.05 shown in red font. (**B**) Heatmap of correlations between repeats and genomic groups (species). Color indicates the sign and magnitude of Spearman’s ρ; bold font and the symbol (*) denote significant associations (*p* < 0.05), and the symbol (†) denotes trends (0.05 ≤ *p* < 0.1).

To identify groups of repeats with similar copy-number profiles across the 14 species (15 samples) studied, a Spearman correlation analysis was performed based on the log-transformed relative copy-number values of the 16 satellite repeat clusters (E1–E16). Coefficients with |ρ| > 0.514 were considered significant, corresponding to *p* < 0.05 at *n* = 15 (the critical value for Spearman rank correlation). The resulting correlation matrix (Table S7) and its visualization as a correlogram (Figure 2A) allowed several groups of repeats with strong positive associations to be identified.

The first group—the most consolidated group—comprises repeats E1, E5, E7, E8, E9, E14 and E15. Correlation coefficients among them range from 0.53 to 0.88, with the highest values noted for the pairs E1–E7 (0.88), E5–E7 (0.87) and E8–E14 (0.87). This group is characterized by concerted changes in copy number across all species studied, suggesting similar regulatory mechanisms or a shared evolutionary dynamic for these repeats.

The second group includes repeats E4, E6, E10, E11 and E12, which also show significant positive correlations, although less tight. The strongest association within this group is observed between E6 and E11, and between E6 and E9 (ρ = 0.75), which links this group to the first group through E9. E4 correlates with E1 (0.55), E10 (0.55), E11 (0.55) and E12 (0.6), indicating an intermediate position of these repeats between the two main clusters.

Repeat E3 does not belong directly to the first group but shows significant correlations with E7 (0.64), E8 (0.61), E9 (0.61) and E14 (0.56), allowing it to be assigned to the same network, albeit with less pronounced similarity.

Distinct patterns were revealed for E2, E13 and E16. E2 and E16 are linked by a positive correlation (ρ = 0.66), while E2 correlates negatively with E12 (ρ = −0.6), indicating opposite trends in the accumulation of these repeats among species. Repeat E13 showed no significant associations with any of the other clusters, suggesting that its copy-number pattern is independent of the other elements studied.

Correlation analysis of the copy numbers of the 16 repeats with binary indicators of the presence of the St, H, Y and V genomes revealed a number of statistically significant associations (*p* < 0.05) (Figure 2B; Table S8). The strongest association was found for repeat E7, whose copy number correlated positively with both the St genome (Spearman’s ρ = 0.54, *p* = 0.040) and the Y genome (ρ = 0.55, *p* = 0.036). Repeat E9 also showed a significant positive association with the St genome (ρ = 0.57, *p* = 0.028).

Repeat E10 showed a significant positive correlation with the H genome (ρ = 0.59, *p* = 0.021), as well as a trend toward an association with the St (ρ = 0.47, *p* = 0.075) and Y genomes (ρ = 0.50, *p* = 0.058).

For E2, a significant negative association with the H genome was found (ρ = −0.59, *p* = 0.021), together with a positive trend with the V genome (ρ = 0.50, *p* = 0.058). A similar negative association with the H genome was obtained for repeat E13 (ρ = −0.59, *p* = 0.021), which, combined with the data for E2, points to a possible opposite directionality in the copy number of these repeats relative to the presence of the H genome.

Several other repeats showed non-significant but notable trends (0.05 ≤ *p* < 0.1): E1 with the St and Y genomes, and E3 and E9 with the Y genome.

Overall, the largest number of significant associations was noted for the St and H genomes (2 and 3 repeats, respectively), whereas no significant associations with repeat copy number were found for the V genome; for the Y genome, significant associations were limited to repeat E7.

#### 2.2.2. Principal Component Analysis of Satellite Repeat Copy-Number Profiles

**Figure 3:**
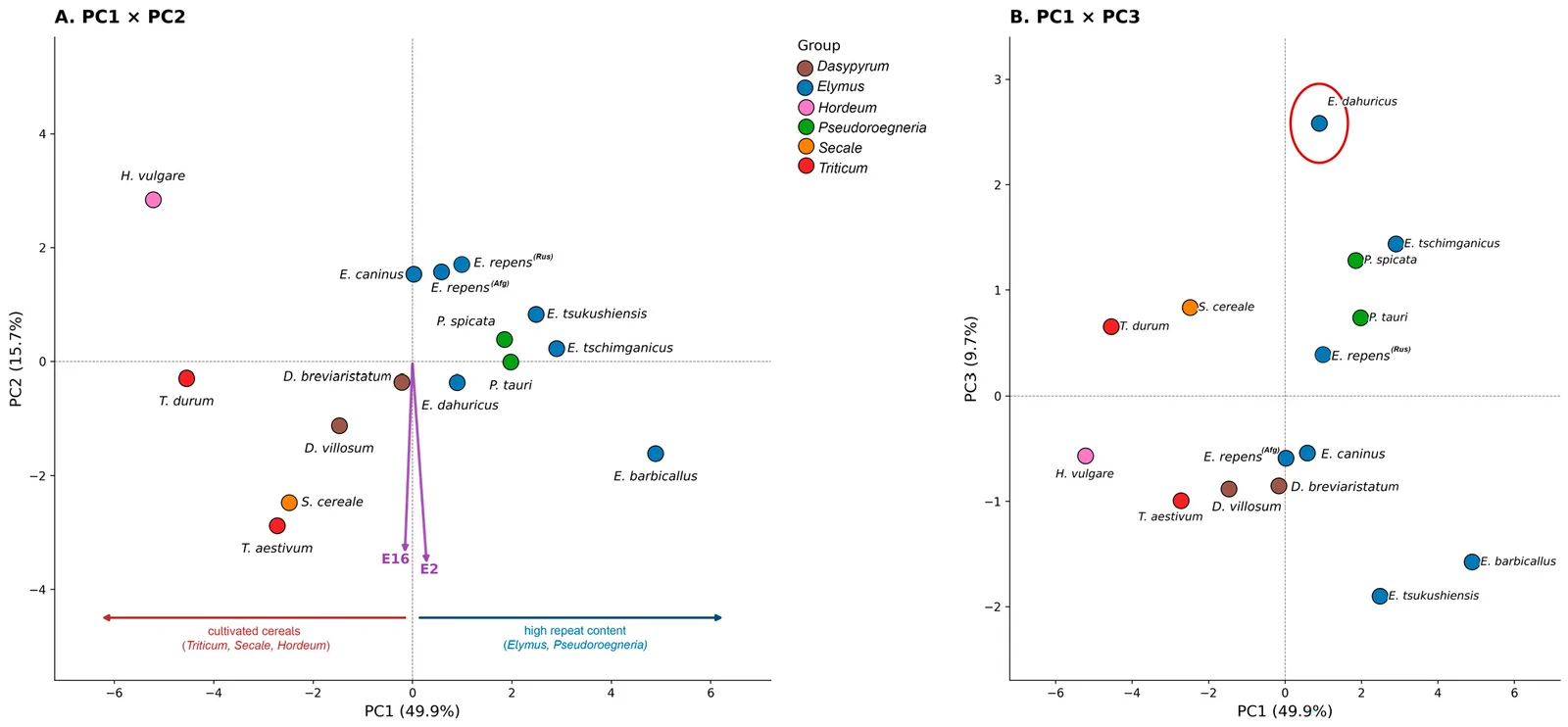
Principal component analysis (PCA) of satellite repeat cluster copy-number patterns in the studied species. Colors indicate taxonomic groups; percentages in parentheses indicate the proportion of variance explained: (**A**) Biplot of species projections on the PC1 and PC2 plane. (**B**) Biplot of species projections on the PC1 and PC3 plane.

To visualize the similarity of copy-number profiles among species, a principal component analysis (PCA) was performed based on the log-transformed relative copy-number values of the 16 repeats. The first two principal components explained 65.3% of the total variance (PC1—49.9%, PC2—15.7%), indicating that the two-dimensional projection is highly informative for describing the data structure (Figure 3A).

PC1 clearly separates the studied species into two main groups (Table S9). Positive PC1 values (from 0.03 to 4.89) are characteristic of perennial forms—species of the genus *Elymus* (*E. tschimganicus*, *E. tsukushiensis*, *E. dahuricus*, *E. caninus*, *E. repens*, *E. barbicallus*), and *Pseudoroegneria* (*P. spicata* and *P. tauri*). Negative PC1 values (from −0.18 to −5.22) are shown by the annual cultivated species—*T. aestivum, T. durum, S. cereale, H. vulgare*—as well as by the diploid *Dasypyrum* species. Thus, PC1 reflects the fundamental distinction between perennial polyploid grasses and annual di- and tetraploid species, consistent with their evolutionary divergence.

Analysis of variable loadings on PC1 showed that almost all studied repeats had positive coefficients (from 0.04 for E2 to 0.33 for E7), with the exception of E16, whose loading was close to zero (−0.02) (Table S10). The largest contributions to PC1 were made by E7 (0.33), E6 (0.30), E5 (0.30), E15 (0.30), E9 (0.30) and E1 (0.31), whereas E2 (0.04), E12 (0.19) and E13 (0.14) had minimal loadings. This allows PC1 to be interpreted as an axis of overall genomic enrichment in satellite repeats: species with positive values (*Elymus, Pseudoroegneria*) have a higher total content of most satellite clusters compared with species of *Triticum, Secale, Hordeum* and *Dasypyrum*. The negative pole of PC1 thus corresponds to genomes in which satellite repeats are represented to a lesser extent, which may reflect both phylogenetic differences and features of genomic organization in cultivated cereals.

The second component (15.7% of the variance) differentiates species within the identified groups and, according to its loadings, contrasts repeats with positive and negative weights. The highest positive loadings on PC2 were found for clusters E12 (0.36), E5 (0.22), E1 (0.10), E7 (0.10) and E11 (0.09), whereas pronounced negative loadings were shown by E16 (−0.51), E2 (−0.54) and E3 (−0.34) (Table S10). Thus, PC2 separates samples according to the ratio of two groups of repeats: on one side, E12, E5, E1, E7 and E11 (positive pole), and on the other, E16, E2 and E3 (negative pole). Species located in the positive PC2 range (*E. caninus*, *E. repens*, *E. tsukushiensis*, *H. vulgare*) are characterized by elevated copy numbers of E12, E5 and E1 and reduced copy numbers of E16 and E2, whereas the opposite pattern is observed in the negative range (*T. durum*, *P. tauri*, *P. spicata*, *E. barbicallus*, *D. breviaristatum*).

It was noted that cluster E16, which has a near-zero loading on PC1, is the main driver of PC2 (loading −0.51; Table S10), indicating a unique distribution pattern unrelated to the overall level of satellite DNA. This is consistent with the results of the correlation analysis (Section 2.2.1), where E16 also showed an isolated pattern.

PC3 explains 9.7% of the variance and, according to its loadings, is determined mainly by E8 (0.47), E4 (−0.52) and E14 (0.36). This component further differentiates species within the clusters, in particular separating *E. dahuricus* (Figure 3B), which may reflect species-specific variation of individual repeats.

#### 2.2.3. Results of Hierarchical Clustering of Species Based on Satellite Repeat Copy-Number Profiles

Hierarchical clustering of samples using Ward’s method based on a matrix of Euclidean distances (Table S11) demonstrated a high level of agreement with the underlying data structure. The cophenetic correlation coefficient was 0.85, indicating good correspondence between the dendrogram and the pairwise distance matrix and high reliability of the resulting cluster structure.

**Figure 4:**
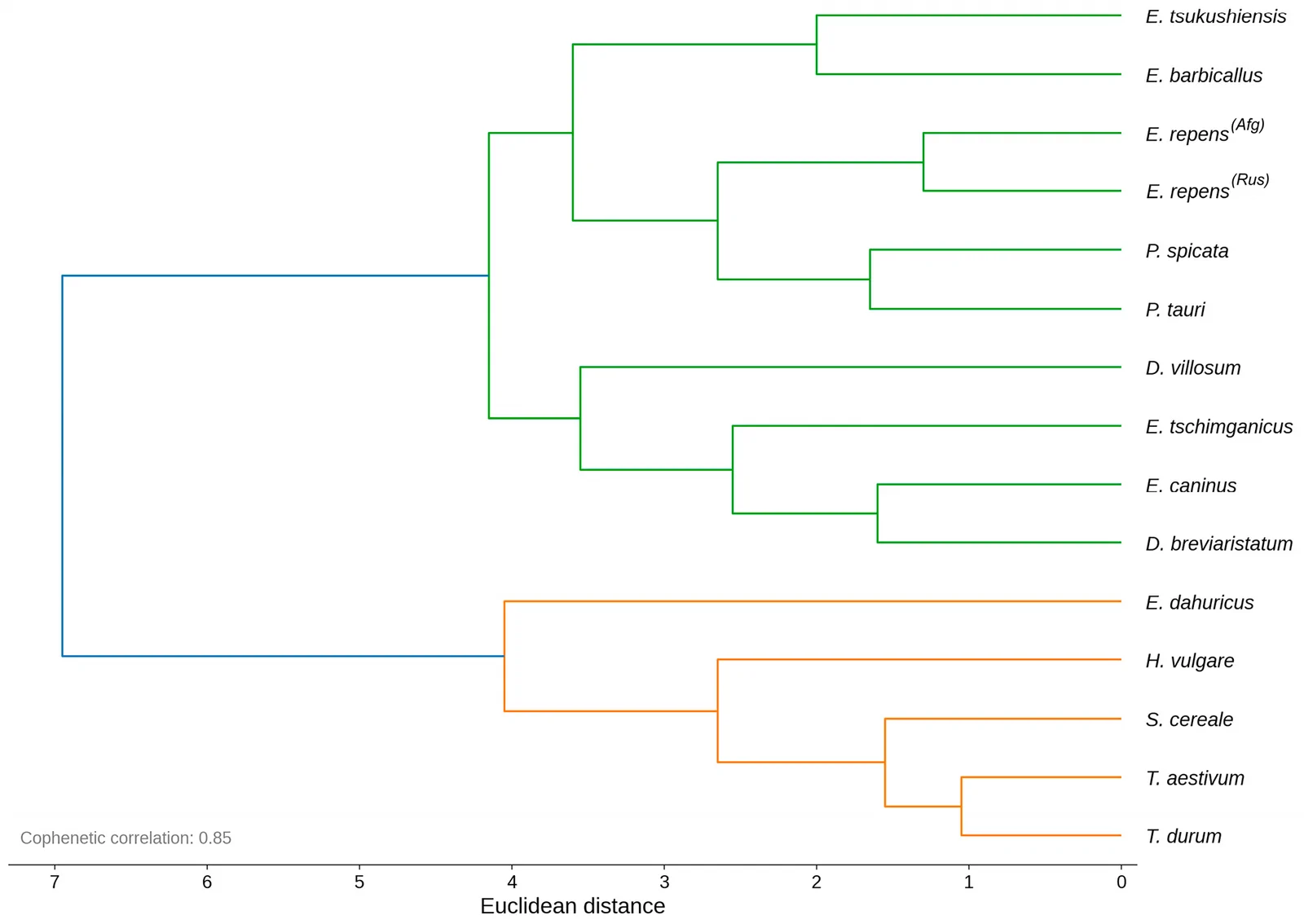
Dendrogram of pairwise Euclidean distances (Ward’s method, repeat copy number).

The resulting dendrogram (Figure 4) grouped the studied species and accessions according to their genomic composition, confirming phylogenetic relationships within the tribe *Triticeae*. Analysis of the cophenetic matrix showed that the minimum cophenetic distance (1.29) was observed between the Russian and Afghan accessions of *E. repens*, indicating a high degree of similarity between geographically distinct accessions of the same species. The second closest pair was formed by *P. spicata* and *P. tauri*, with a Euclidean distance of 1.65, indicating a high degree of similarity in the traits studied. These pairs joined at the lowest levels of clustering, reflecting their relatively high similarity compared with the other taxa included in the analysis.

The dendrogram structure also revealed stable clusters uniting species with smaller interspecific distances. The main clades had bootstrap support above 85%, indicating the reproducibility of the resulting topology upon resampling of the data.

The dendrogram reflects the formation of several well-separated clusters corresponding to the taxonomic affiliation of the species. The most isolated position is occupied by *E. dahuricus*, which is characterized by nearly equal cophenetic distances relative to all other taxa (approximately 7.13). Such equidistance may indicate an intermediate position of this taxon, a possible hybrid origin, or the need for additional verification of its taxonomic identification using independent molecular-genetic markers.

*H. vulgare* forms a separate branch of the dendrogram, reflecting its considerable genetic distinctness from representatives of the genus *Elymus* and related taxa.

Thus, the dendrogram constructed by Ward’s method reflected the heterogeneity of the studied set and revealed both closely related taxa or accessions and more distinctly separated members of the cluster structure. The closest relationship was observed between the Afghan and Russian accessions of *E. repens*, followed by *P. spicata* and *P. tauri*. In contrast, *E. dahuricus* and *H. vulgare* howed the greatest degree of separation from the other taxa included in the analysis.

#### 2.2.4. Copy-Number Assessment and FISH Localization of Repeats E6 and E11 in *Triticum aestivum*–*Dasypyrum villosum* Lines

Interspecific correlation analysis did not reveal statistically confirmed V-genome associations owing to the limited representation of V-containing taxa; however, a direct comparison of *T. aestivum* and *D. villosum*, followed by analysis of introgression lines, allowed the selection of clusters E6 and E11: low levels in bread wheat (E6—1.31; E11—2.50) and high levels in *D. villosum* (E6—4.98; E11—4.97). This contrast provided the basis for their selection as potential V-subgenome candidates and for further testing on a series of *T. aestivum*–*D. villosum* addition lines.

The copy number of clusters E6 and E11 was further assessed by qPCR in disomic *T. aestivum*–*Dasypyrum* addition lines carrying individual added *D. villosum* chromosomes: W1 (1V), W3 (3V), W4 (4V), W5 (5V), W6 (6V) and W7 (7V). The results, normalized to the reference gene *VRN1* and expressed as the decimal logarithm of relative copy number, are presented in Table 1.

The copy number of repeat E6 in bread wheat remained low (1.31). In three addition lines—W3 (3V), W4 (4V) and W7 (7V)—a marked increase in copy number was observed, reaching 2.22, 2.39 and 2.74, respectively. In lines W1 (1V), W5 (5V) and W6 (6V), the copy number of E6 (1.14–1.39) was virtually indistinguishable from that of bread wheat. This distribution indicates the presence of copies of cluster E6 on several *D. villosum* chromosomes simultaneously—3V, 4V and 7V—with maximal accumulation on chromosome 7V, whereas chromosomes 1V, 5V and 6V appear not to be enriched for this repeat.

**Figure 5:**
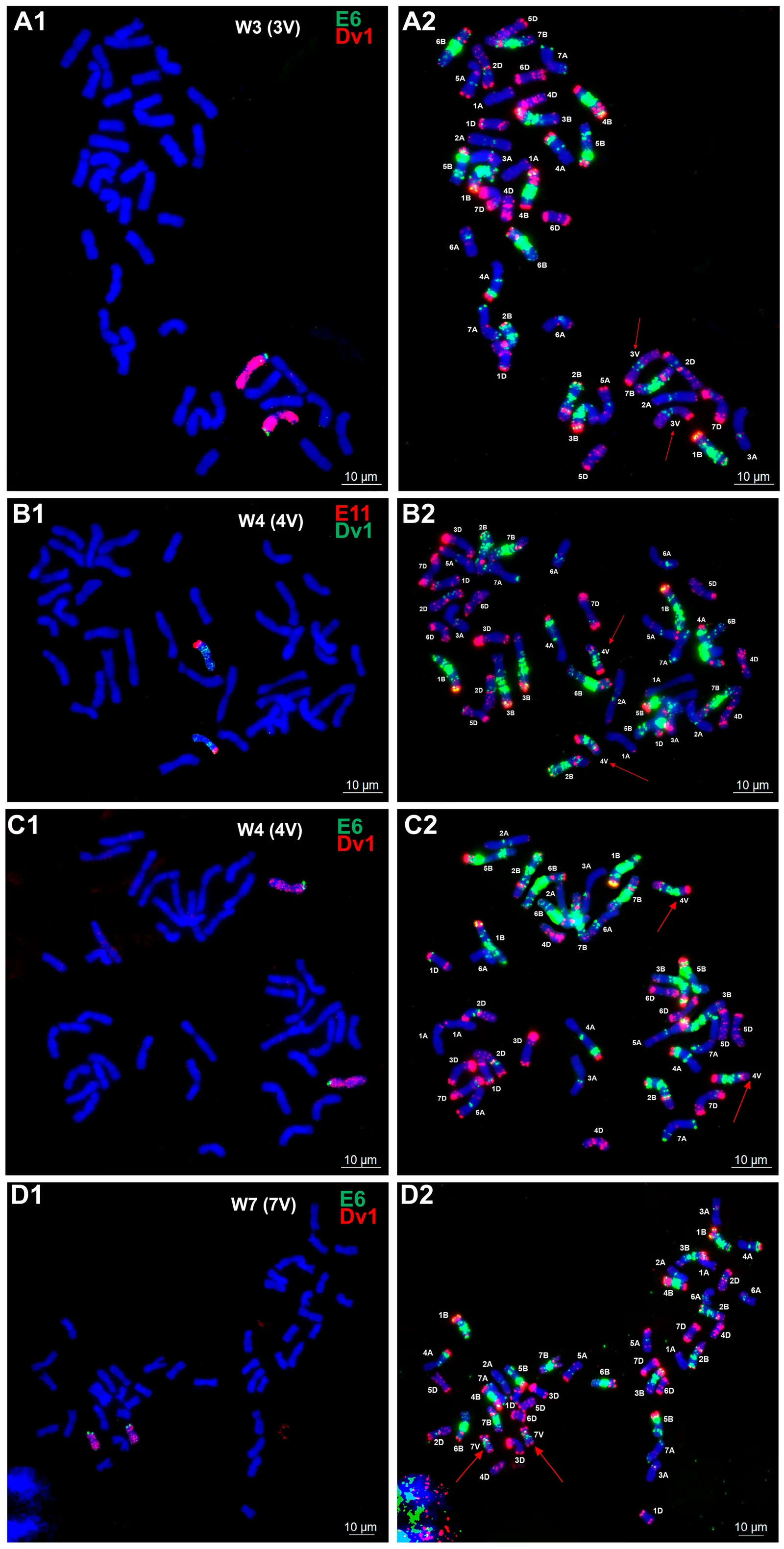
FISH results with probes E6 and E11 on metaphase chromosomes of CS-*Dasypyrum* addition lines: (**A1**,**A2**)—line W3 (3V), probe E6 (green) and Dv1 (red); (**B1**,**B2**)—line W4 (4V), probe E11 (red) and Dv1 (green); (**C1**,**C2**)—line W4 (4V), probe E6 (green) and Dv1 (red); (**D1**,**D2**)—line W7 (7V), probe E6 (green) and Dv1 (red). Images 1—combination of probes E6 and Dv1, E11 and Dv1; images 2—the same metaphase plates after FISH with oligo-pAs1-1 + oligo-pSc119.2-2 (red), GAA_n_ (green); wheat chromosomes are labeled by homoeologous group number and subgenome (A, B, D), and arrows indicate the added/substituting V chromosomes of *D. villosum*. Chromosomes counterstained with DAPI (blue). The bar indicates 10 µm.

For repeat E11, the pattern was fundamentally different: copy number was low in bread wheat and in most addition lines. The only line showing a sharply pronounced amplification of repeat E11 was W4 (4V). These data indicate a predominant or exclusive localization of the main pool of E11 copies on chromosome 4V of *D. villosum*. Based on the qPCR results, lines W3, W4 and W7 were selected for cytogenetic verification of E6 localization, and line W4 for verification of E11. FISH analysis confirmed the localization of both repeats on V-genome chromosomes (Figure 5). The described FISH signal patterns were consistently observed in all analyzed metaphase plates, with at least 10 metaphase plates examined per preparation. In line W3, probe E6 produced terminal signals on a pair of 3VL chromosomes (Figure 5A1). Subsequent karyotyping with known oligo-probes showed the absence of the wheat 3D chromosome pair (Figure 5A2), indicating that line W3 was in fact the 3V(3D) substitution line.

Line W4 was characterized by terminal E11 signals on chromosome 4VL (Figure 5B1), as well as terminal E6 localization on the same chromosome pair, but on the short arm (Figure 5C1). Karyotyping revealed the absence of the 4B pair, allowing W4 to be characterized as the 4V(4B) substitution line (Figure 5B2,C2).

On the chromosomes of line W7, cluster E6 produced terminal signals on chromosome 7VS (Figure 5D1). Unlike W3 and W4, the wheat chromosome complement in W7 was retained, with 7V chromosomes present as an additional pair (Figure 5D2).

Thus, FISH localization fully confirmed the results of the preliminary qPCR screening: E6 was localized on chromosomes 3V, 4V and 7V, and E11 on 4V. These findings confirm the suitability of E6 and E11 as V-chromosome-associated FISH markers; E11 shows a narrower specificity to 4V, whereas E6 marks several V-genome chromosomes. At the same time, cytogenetic analysis clarified the karyotypic status of the material: W3 and W4 are the 3V(3D) and 4V(4B) substitution lines, respectively, whereas W7 remains a 7V addition line.

## 3. Discussion

The combined data on the homology and taxonomic distribution of satellite repeats E1-E16, their relative copy number in Triticeae representatives, and the multivariate structure of the observed variation allow several interrelated aspects to be discussed. First, comparison of the results of bioinformatic analysis, qPCR, correlation analysis, PCA and hierarchical clustering makes it possible to assess the degree of conservatism of the repeats, the features of their amplification, and the relationship between copy-number profiles and the generic affiliation and genomic composition of the species studied. Second, on this basis, repeats potentially associated with the St, Y and H genomic components can be identified, and the limitations of using statistical associations for selecting subgenome-specific FISH probes can be assessed. Third, the features of the repeatome profiles and the position of the *E. dahuricus* and *E. barbicallus* samples in the multivariate analyses allow possible reasons for the discrepancy between their actual chromosome number and literature data to be considered, including intraspecific variability, aneuploidy/disploidy, reticulate evolution, and possible hybrid origin. Finally, the results of qPCR screening and FISH localization of repeats E6 and E11 allow their suitability as markers of individual V chromosomes of *D. villosum* to be assessed, the karyotypic status of *T. aestivum*–*Dasypyrum* lines to be clarified, and possible changes in their chromosomal composition during prolonged propagation to be discussed.

### 3.1. Common and Specific Repeats: Consistency of Homology, Copy-Number and Statistical Data

Comparison of homology, relative copy-number and statistical structure data showed that the identified satellite repeats E1–E16 differ in their degree of conservatism and association with individual genomic components. This result is consistent with the concept of satellite DNA as a dynamic genomic fraction characterized by rapid changes in copy number, amplification of certain families, and their reduction or redistribution among chromosomes during plant evolution and polyploidization [6,14,26]. Homology reflects the origin or structural relatedness of the repeats, presence/absence data reflect their distribution among the taxa studied, qPCR characterizes the level of amplification, and correlation analysis and PCA allow the identification of repeats with similar copy-number dynamics.

Overall, the repeats can be divided into several groups: widely distributed Triticeae repeats, repeats showing statistical associations with St/StY genomic backgrounds, elements with limited or non-standard distribution, and repeats potentially linked to the H and V components. At the same time, statistical analysis shows that these groups do not fully coincide with groups defined solely on the basis of homology. This is important, since a widely distributed repeat may show independent copy-number dynamics, while a repeat with a more limited distribution may covary with the general pool of St-associated sequences.

The first group comprises repeats with a wide distribution and pronounced homology to previously described sequences from various Triticeae genera. E2, E4, E6 and E7 are the most representative examples in this respect. These repeats show similarity to satellite or FISH-positive sequences of *Triticum*, *Secale*, *Hordeum*, *Aegilops*, *Thinopyrum* and *Pseudoroegneria*, indicating their affiliation with a conserved pool of repetitive sequences within the tribe. Such wide intergeneric similarity is consistent with data indicating that Triticeae satellite repeats can be both highly specific and represented across different genera, while retaining different levels of copy number and chromosomal localization [9,25,27,28].

However, correlation analysis data show that repeats within this general group do not behave uniformly. The most consolidated correlation group (clusters E1, E5, E7, E8, E9, E14 and E15), in which correlation coefficients between clusters reach high values, especially for the pairs E1–E7, E5–E7 and E8-E14, largely coincides with the repeats that contribute most strongly to PC1 in the PCA—the axis separating perennial St-containing taxa (*Elymus*, *Pseudoroegneria*) from annual or more distantly related genera (*Triticum*, *Secale*, *Hordeum*, *Dasypyrum*). Therefore, the first correlation group can be regarded as a set of repeats whose copy number changes in a concerted manner and that make the main contribution to the overall difference between St-containing polyploid species and the remaining representatives of the sample.

Cluster E7 is particularly informative within this group. On the one hand, it is a widely distributed repeat, as it was identified in all studied representatives of *Elymus*. On the other hand, its closest homologs were found among satellites of *P. tauri* and *P. spicata* (St genome), and its copy number correlates positively with the presence of the St and Y genomes. In addition, E7 belongs to the first correlation group and has a high contribution to PC1, confirming its association with the general pattern of repeat accumulation in St-containing taxa. Thus, E7 should not be considered a St- or Y-specific marker, but rather a putative candidate whose copy-number variation is statistically associated with the presence of the St and Y genomic components. This interpretation is consistent with data on the key role of *Pseudoroegneria* representatives as St-genome donors for many *Elymus* species [29,30].

Interestingly, the clustering of satellite repeat copy-number profiles also revealed an affinity between *Dasypyrum* and several polyploid *Elymus* representatives. Although repeatome-based clustering alone cannot establish direct genome ancestry, this pattern is consistent with recent chromosome-scale genomic studies indicating a close evolutionary relationship between the Y haplome of polyploid *Elymus* sensu lato and the V lineage of *Dasypyrum*. Xiong et al. [31] showed that the Y haplome of allohexaploid *E. nutans* is sister to the clade comprising the V haplome of *Dasypyrum* and the J^v^ haplome of *Thinopyrum*. Similarly, Sun et al. [32] demonstrated that the Y-related subgenome of *Roegneria kamoji* (Ohwi) Ohwi ex Keng belongs to an evolutionarily divergent lineage closely related to the V clade of *Dasypyrum*. Thus, the similarity in satellite repeat profiles observed here may reflect this deeper evolutionary relationship between the Y and V genomic lineages rather than direct evidence of contribution from an extant *Dasypyrum* genome.

At the same time, not all widely distributed repeats belong to the same correlation block. For example, E2 is a widely distributed and high-copy repeat, but its level varies noticeably among species and does not form as stable a correlation block as E7 or E14. This means that E2 reflects the conservatism of the repeat family rather than concerted amplification within a single genomic group. E4, by contrast, combines wide homology, high mean copy number, and low interspecific variability, but statistically belongs not to the first but to the second correlation group, together with E6, E10, E11 and E12. Consequently, E4 can be regarded as a stable, widely distributed, high-copy repeat, but its quantitative dynamics are closer to a different repeat block. A similar discrepancy between homology, copy number and cytogenetic detectability has previously been shown for satellite sequences of *Ae. crassa* and related Triticeae, where homologous repeats differed in their level of representation, FISH signal intensity, and localization among species [9].

The second correlation group includes E4, E6, E10, E11 and E12. It is less homogeneous in biological meaning than the first: it includes both widely distributed repeats, E4 and E6, and elements with a more pronounced association with particular genomic backgrounds, such as E10, E11 and E12. This indicates that similar copy-number dynamics do not always imply common origin or identical genomic specificity. Rather, this group reflects a combination of two processes: general accumulation of high-copy repeats and local amplification of individual repeat families in particular lines [33,34]. Also important within this group is E10, since it showed a significant positive correlation with the presence of the H genome. Its homology relationships are not limited to *Hordeum* alone, but it is precisely its copy-number profile that makes E10 the most convincing candidate for the H component. E12, in contrast, has its closest homologs among *H. vulgare* sequences, but does not show H-specificity in terms of copy number and belongs to a broader correlation block. This underscores that homology with *Hordeum* alone is not sufficient grounds for classifying a repeat as an H-specific marker: consistency among homology, copy number, statistical association with the genome, and further FISH validation is required [9,18].

Several repeats showed copy-number patterns associated with St-containing genomic backgrounds, including E9 and, to a lesser extent, E13 and E14. E9 showed a significant positive association with the St genome and belongs to the first correlation group, indicating its concerted dynamics with other St-associated repeats. However, its distribution across the comparative assemblies and the extended sample was not entirely unambiguous, so E9 should be regarded as a promising but not yet fully verified St-associated candidate. E13 and E14 show similarity to repeats of *P. libanoticus* and a number of Triticeae sequences, allowing them to be regarded as a closely related repeat block associated with St-containing or closely related genomic backgrounds. At the same time, E14 belongs to the first correlation group and correlates closely with E8, whereas E13 shows no significant associations with the other repeats. This is an important distinction: homologically related repeats can display different copy-number dynamics, which is typical of satellite DNA, whose evolution is determined not only by sequence origin but also by subsequent independent amplification or elimination [6,26].

Repeats with limited or non-standard distribution are represented by clusters E3, E5, E15 and E16. E3 and E5 were not found in all species studied but have homologs among satellite sequences of other Triticeae genera. This indicates not their absolute uniqueness, but rather selective retention, reduction, or amplification in particular genomic backgrounds. This pattern is consistent with the satellite DNA “library” model, according to which related species may share a common set of repeat families but differ in their level of representation in the genome [6,33]. At the same time, E5, despite its limited distribution, belongs to the first correlation group and has a high contribution to PC1. Consequently, a limited presence of a repeat does not preclude its concerted copy-number dynamics with the general St-associated pool where it is present. E15 and E16 differ from the other repeats in that they were identified through comparison of *E. barbicallus*, *E. pendulinus* and *E. arizonicus* and were absent in the other species. E15 shows similarity to several classes of mobile elements from different genera, including *A. cristatum*, *Ae. tauschii* and *T. monococcum*, which may indicate its association with transposon or retrotransposon sequences [2,35,36]. At the same time, E15 belongs to the first correlation group and makes a notable contribution to PC1, making it an example of a repeat with limited distribution but concerted quantitative dynamics with the general St-associated block. E16, in contrast, has no annotated homologs in NCBI, is characterized by low copy number and an isolated statistical pattern, and is one of the main drivers of PC2. E16 can therefore be regarded as a potentially new repeat with independent dynamics, although its practical value as a FISH marker requires separate verification.

Interspecific correlation analysis did not reveal statistically confirmed V-genome associations, which was attributable to the limited representation of the V genome among the taxa studied.

Thus, this combination of homology, copy number and statistical associations allows several candidate repeat groups to be identified for subsequent FISH validation.

1. Cluster E7 represents a putative candidate whose copy-number variation is statistically associated with the St and Y genomic components. Its copy number correlates significantly and positively with two subgenomes simultaneously: St (ρ = 0.54; *p* = 0.040) and Y (ρ = 0.55; *p* = 0.036). This dual association may have several explanations. First, E7 may be present in both the St and Y subgenomes, i.e., it may mark not a single but a shared St/Y-associated repeat component. Second, the positive association with two subgenomes may reflect a close evolutionary history of the St and Y components in *Elymus*, since the origin of the Y genome remains debated [24,30], and part of the repeat may be common to the St and Y subgenomes. Third, this correlation may result from the joint presence of St and Y in several polyploid taxa of the sample. E7 should therefore not be interpreted as a strictly St- or strictly Y-specific marker. It is therefore more appropriate to regard E7 as a candidate for further cytogenetic testing of its putative association with St/StY genomic backgrounds.
2. Cluster E9 represents a putative candidate statistically associated with the St genomic component, since its copy number showed a significant positive correlation with the presence of the St genome (ρ = 0.57; *p* = 0.028). Its association with the Y genome was observed only as a trend, and E9 therefore cannot be regarded as a Y-specific marker. The putative association of E9 with St/StY genomic backgrounds should be independently validated by FISH using a broader panel of species with known St, StY, StH and StHY genomic compositions.
3. For the H component, the most well-founded candidate is E10. Its copy number correlates significantly and positively with the presence of the H genome (ρ = 0.59; *p* = 0.021), and also shows a trend toward a positive association with the St genome (ρ = 0.47; *p* = 0.075). This pattern may indicate two possible scenarios. In the first, E10 is predominantly associated with the H subgenome but is also partly represented in St-containing genomes. Alternatively, cluster E10 may mark not a strictly H-specific repeat but a repeat component characteristic of StH genomic combinations. E10 should therefore be regarded as a statistically supported candidate associated with the H genomic component rather than as an H-specific marker, and its specificity requires independent cytogenetic validation.

Thus, the associations of E7, E9 and E10 inferred from copy-number correlations should be regarded as preliminary, and chromosome or subgenome specificity cannot be established without independent cytogenetic validation across a broader taxonomic panel.

### 3.2. Atypical Chromosome Numbers and Repeatome Profiles of E. dahuricus PI 634270 and E. barbicallus PI 504441

For *E. dahuricus*, the genomic formula StHY (2n = 42) is generally accepted in the literature and has been repeatedly confirmed by GISH and chromosome-painting methods [37,38]. The accession studied here (PI 634270) has 2n = 28, which precludes assigning it the standard hexaploid formula. Chromosome counts were performed using three independently grown plants of this accession, and the same plants were used as biological replicates for qPCR-based repeat copy-number assessment. This discrepancy may have several explanations, which are not mutually exclusive.

First, *E. dahuricus* in the broad sense represents an aggregate species, within which at least two cryptic taxa have been identified on the basis of ITS rDNA polymorphism [39]. If PI 634270 belongs to one of these divergent lineages, its deviation from the typical hexaploid formula, up to a different ploidy level, would not contradict the known intraspecific variability of the genus *Elymus*.

Second, *Elymus* is characterized by considerable reticulate evolution and recurrent interspecific and intraspecific hybridization [40]. Therefore, the atypical chromosome constitution of PI 634270 may reflect hybridization involving genetically differentiated forms or cytotypes within the reticulate *Elymus* complex, followed by subsequent chromosome restructuring. Third, *E. dahuricus* is characterized by numerous intergenomic translocations (mainly H/Y and H/St) [37], which do not by themselves change the chromosome number, but in combination with Robertsonian fusions or other dysploid events known in polyploid Triticeae, may lead to a secondary reduction in chromosome number without complete loss of genetic material from the corresponding subgenomes.

Our data on satellite repeats also indicate that accession PI 634270 differs from typical representatives of *E. dahuricus*. This accession lacks repeat E4, which is a high-copy, near-universal cluster among all six main taxa. In addition, cluster E9—a putative St/StY-associated repeat—was not detected in it. Finally, on the dendrogram and in the PCA, PI 634270 occupies a statistically isolated position, differing from all other samples. The karyotype and repeat profile of PI 634270 do not correspond to the typical hexaploid StHY form of *E. dahuricus*. The reasons for this discrepancy remain unclear and may include intraspecific cytotype variability, aneuploidy or dysploidy, hybrid origin, reticulate evolution, or spontaneous hybrid origin involving genetically differentiated forms or cytotypes. Accordingly, no definitive genomic formula is assigned here to PI 634270. Further GISH/FISH analysis will be required to determine its genomic composition and to clarify the cytogenomic basis of its atypical chromosome constitution.

For *E. barbicallus*, the situation also remains ambiguous. The literature reports a formula of StY at 2n = 28 for this species [41], whereas the accession studied here (PI 504441) has 2n = 35. As for PI 634270, chromosome number was assessed in three independently grown plants, which were also used as biological replicates in the qPCR analysis. This deviation may be related to aneuploidy, intraspecific chromosome-number variation, or spontaneous hybrid origin within the reticulate *Elymus* complex. Because its genomic composition has not been independently established, we do not assign a definitive genomic formula to PI 504441 in the present study.

However, the satellite repeat data support retaining PI 504441 in the comparative analysis as an atypical accession. Its copy-number profile retains an association with other representatives of *Elymus*: it is characterized by a high representation of several repeats also amplified in other St-containing *Elymus* taxa, including E3, E7, E13 and E14. Moreover, in the hierarchical clustering by copy number, the most isolated accessions in the sample were *E. dahuricus* and *H. vulgare*, whereas *E. barbicallus* did not form as isolated a branch. This indicates that, despite its non-standard chromosome number, its repeatome profile as a whole remains closer to the St-containing *Elymus* group than to more distantly related Triticeae genera.

Importantly, because *E. dahuricus* and *E. barbicallus* are each represented by a single accession in the present study, the observed chromosome-number and repeatome patterns are interpreted at the accession level and should not be generalized to the species as a whole. These accessions were retained in the comparative analyses because they provide information on cytogenomic and repeatome variation observed within the *Elymus* complex; however, conclusions concerning their precise genomic composition remain provisional pending further cytogenetic characterization.

### 3.3. Validation of V-Chromosome-Associated Repeats E6 and E11 and Karyotypic Variability of T. aestivum–D. villosum Lines

The concordance between qPCR and FISH results confirmed the effectiveness of the preliminary selection of repeats by copy number. Repeat E6 was localized in the terminal regions of chromosomes 3VL, 4VS and 7VS, whereas E11 was localized to 4VL. This difference corresponds to the well-known uneven distribution of satellite repeats among chromosomes of a single genome in Triticeae [6,26]. Similar heterogeneity has previously been shown in *Dasypyrum*, where some repeats are distributed across several chromosomes while others are restricted to a single chromosome pair [25]. Comparison with the *Dasypyrum* satellite repeats characterized previously [25] revealed 96.25% sequence identity between E6 and CL169 and 74.27% identity between E11 and CL135. In *D. villosum* W6 21717, CL169 produced terminal signals on all chromosomes, whereas in *D. breviaristatum* PI 516547 it showed terminal and/or proximal localization on several V and Vᵇ chromosomes. In contrast, CL135 was not detected by FISH in *D. villosum* but produced terminal signals on 1VS, 4VS and 6VL in *D. breviaristatum*. The high similarity between E6 and CL169 is consistent with their predominantly terminal localization, whereas the substantially lower similarity between E11 and CL135 may partly explain their contrasting FISH patterns and indicates considerable divergence of these repetitive sequences. Consequently, E11 can be regarded as a more specific marker of chromosome 4V, whereas E6 serves as a marker of several V chromosomes. These results are consistent with the use of repetitive sequences for identifying *D. villosum* chromosomes on a wheat genomic background and confirm the utility of combining qPCR screening with subsequent FISH validation [9,23].

In addition to confirming the chromosomal affiliation of E6 and E11, FISH analysis with a panel of standard oligonucleotide probes made it possible to clarify the karyotypic status of lines W3 and W4, showing that they are not addition lines but substitution lines—3V(3D) and 4V(4B), respectively. The independent derivation of the 3V(3D) line from the progeny of a cross between Chinese Spring and a 3V addition line has previously been described [42]. In addition, among *T. aestivum*–*D. villosum*, variants of substitution of chromosomes 4B and 4D by chromosome 4V are known [43]. Thus, the status of W3 and W4 identified here is biologically plausible and highlights the need for repeated karyotyping of introgression lines after cycles of prolonged propagation.

The transition from an addition to a substitution karyotype can also be interpreted as one of the possible pathways for stabilizing chromosomal composition. Disruptions of meiotic pairing and gradual elimination of individual chromosomes over successive generations have been demonstrated for alien introgressions in wheat [44]. Under these conditions, retention of the V-chromosome pair with loss of the homoeologous wheat chromosome pair could result in a more balanced complement of 42 chromosomes and be maintained by selection during propagation.

## 4. Materials and Methods

### 4.1. Plant Material

The study used representatives of the tribe Triticeae spanning six genera with different genomic compositions: *Elymus*, *Dasypyrum*, *Pseudoroegneria*, *Triticum*, *Hordeum* and *Secale*. A list of the accessions studied, with species name, accession number, ploidy, genomic formula, purpose of use, and source, is given in Table 2.

The copy-number study also included *T. aestivum*–*D. villosum* addition lines, kindly provided by Adam J. Lukaszewski: W1 (chromosome 1V), W3 (chromosome 3V), W4 (chromosome 4V), W5 (chromosome 5V), W6 (chromosome 6V) and W7 (chromosome 7V). Analyses were performed using seed material obtained after several successive generations of line propagation under collection-maintenance conditions.

### 4.2. Sequencing and Bioinformatic Processing

Freshly cut young leaves were ground in liquid nitrogen and genomic DNA was extracted following the CTAB protocol [46]. DNA was used for genome sequencing, quantitative PCR, and synthesis of DNA probes for FISH. The quantity and quality of the extracted DNA were verified using a NanoDrop OneC spectrophotometer (Thermo Fisher Scientific, USA). DNA samples were considered of adequate quality if the A260/280 ratio ranged from 1.8 to 2.0 and the A260/230 ratio from 2.0 to 2.2. DNA concentration was measured using a Qubit 4 instrument with Qubit™ dsDNA HS and BR Assay Kits (Thermo Fisher Scientific, Waltham, MA, USA).

Whole-genome shotgun libraries were prepared from 25 ng of genomic DNA using the Swift 2S^®^ Turbo DNA Library Kit (Swift Bioscience, Ann Arbor, MI, USA) according to the manufacturer’s protocol, with a target fragment size of approximately 350 bp. Libraries were indexed using the Swift 2S Turbo Unique Dual Indexing Kit. Sequencing was performed on an Illumina NextSeq platform (Illumina, Inc., San Diego, CA, USA) using the NextSeq 500/550 Mid Output Kit v.2.5 (Illumina, Inc.) in paired-end mode, generating 2 × 155 bp reads.

The quality of the raw paired-end reads was assessed using FastQC v0.12.1 [47]. and adapter sequences and low-quality bases were removed using the BBDuk utility (BBMap package, v39.27; [48]; parameters: ktrim = r, k = 20, mink = 10, hdist = 2). From the filtered reads, a random subsample of 2 × 10^6^ paired reads per sample was generated using a fixed random-number-generator seed to ensure reproducibility of the results.

### 4.3. Identification of Tandem Repeats

Tandem repeats were identified by graph-based clustering using the RepeatExplorer2 pipeline [10] with the TAREAN module [8]. The minimum cluster size considered was 0.01% of the total number of reads, ensuring the detection of both high- and medium-copy elements. Satellite repeats were identified based on the circular topology of cluster graphs, characteristic of tandemly organized sequences. Only high-confidence satellites (TAREAN rank 1) that simultaneously satisfied the following criteria were included in further analysis: probability of satellite nature > 0.7; graph connectivity index C > 0.8; cluster proportion of total reads > 0.1%. For each selected repeat, a consensus monomer sequence was reconstructed.

To search for potential Y-specific clusters, three comparative assemblies were also generated, in which the number of reads for each species was calculated in direct proportion to ploidy level: *E. repens^(Afg)^* + *E. tsukushiensis*, *E. repens^(Rus)^* + *E. tsukushiensis*, and *E. barbicallus* + *E. pendulinus* + *E. arizonicus*.

Homology of the identified repeats to known sequences was established by blastn searches (BLAST+ 2.16.0) against a nucleotide database compiled from NCBI for the taxonomic group Triticeae (ID: 147389), filtered by keywords («satellite», «repeat», «retrotransposon», «marker», etc.) and by sequence length (100–50,000 bp). Searches were performed in three modes: megablast (identity > 95%), dc-megablast (70–95%) and standard blastn (<70%); the E-value threshold was 0.1, and the minimum identity was 60%. Query sequence coverage was calculated by summing the lengths of merged, non-overlapping local alignments (HSPs) and expressed as a percentage of monomer length.

When comparing results across different assemblies, the absence of a repeat among independently identified clusters was not interpreted as complete absence of the corresponding sequence from the genome. Low-copy or structurally divergent variants may not have formed a separate cluster owing to the established representation threshold or features of graph-based clustering. Therefore, the taxonomic distribution of repeats was assessed taking into account both the origin of reads in the comparative assemblies and the similarity of the resulting consensus sequences.

Primers for qPCR were designed based on the consensus monomer sequences of the clusters (Table 3) obtained in TAREAN. Primer design was performed using Primer3 (v2.6) [49].

### 4.4. Real-Time Quantitative PCR

Real-time quantitative PCR (qPCR) was performed using DNA from the species listed in Table 2 as templates, in three biological replicates. The single-copy nuclear gene *VRN1* was used as the reference gene for data normalization. *VRN1* is present in a single copy per haploid genome in diploid Triticeae species and is the most widely used reference for relative qPCR analysis of repetitive sequences within this tribe [19,50]. The qPCR reaction was performed in a volume of 11 μL containing: 5 μL of PCR mixture based on Eva Green qPCR master mix (Syntol Ltd., Moscow, Russia), 10 ng/μL of each primer, and 0.4 ng/μL of genomic DNA. Reactions were set up in 384-well plates on a CFX Real-Time PCR Detection System (Bio-Rad, Hercules, CA, USA) in three technical replicates. Reaction conditions were as follows: initial denaturation at 95 °C for 10 min; followed by 45 cycles of denaturation for 10 s at 95 °C and primer annealing for 30 s at 60 °C.

The relative quantity (RQ) and quantification cycle (Cq) of each satellite repeat were calculated using the ΔΔCt method, with the *VRN1* gene as reference, in Bio-Rad CFX Manager 3.1 software.

### 4.5. Statistical Analysis

The unit of analysis in interspecific comparisons was the species (accession). For each «sample × repeat» combination, the mean Cq value of three technical replicates was used. The relative copy number of the repeats (RQ) was calculated using the ΔΔCt method after normalization to the single-copy gene *VRN1*.

Since RQ values varied over several orders of magnitude, they were transformed using the formula log_10_(RQ). For each repeat, the mean, standard deviation, minimum and maximum were calculated. Interspecific variability was additionally assessed using the coefficient of variation. The coefficient of variation of satellite repeat copy-number values among species was calculated using Microsoft Excel (USA). For repeat E16, whose mean log-transformed value was close to zero, the coefficient of variation was not interpreted.

Similarity of repeat copy-number patterns was assessed using Spearman’s rank correlation coefficient (ρ) based on the log_10_(RQ) matrix for 15 accessions and 16 repeats. Spearman correlations were additionally calculated between the copy number of each repeat and binary indicators of the presence of the St, H, Y and V genomes. Two-tailed *p*-values were used; differences were considered significant at *p* < 0.05, and values of 0.05 ≤ *p* < 0.10 were regarded as trends.

Principal component analysis (PCA) was performed on the correlation matrix of standardized log_10_(RQ) values. Interpretation was based on the proportion of variance explained by the principal components, species coordinates, and repeat loadings. Hierarchical clustering of species was performed using Ward’s method on a matrix of Euclidean distances calculated from the standardized log-transformed data. Agreement between the dendrogram and the original pairwise distances was assessed using the cophenetic correlation coefficient. Cluster stability was evaluated using 1000-fold bootstrap resampling with calculation of branch support.

The qPCR data for the *T. aestivum*–*D. villosum* addition lines were analyzed descriptively by comparing log_10_(RQ) values with the *T. aestivum* (CS) and *D. villosum* controls. Owing to the absence of independent biological replicates, *t*-tests, analysis of variance, and other statistical methods for comparing lines were not applied.

All statistical calculations and visualizations were performed in R version 4.4.

### 4.6. DNA Probes for FISH and Fluorescence In Situ Hybridization

Cytological preparations were made from roots according to the protocol of Badaeva et al. [51]. Repeats E6 and E11 were selected as FISH probes because qPCR revealed a pronounced contrast in their copy numbers between *T. aestivum* and *D. villosum*: both repeats showed low copy numbers in bread wheat and high copy numbers in *D. villosum*. PCR amplification was performed in a 25-μL reaction mixture containing approximately 50 ng genomic DNA, 1.5 μL of 10× PCR buffer, 1.5 mM MgCl_2_, 0.2 mM of dNTPs, 0.3 μM of each primer, and 0.5 unit of *Taq* DNA polymerase. The PCR conditions were as follows: an initial denaturation step of 95 °C for 5 min, followed by 35 cycles of 95 °C for 30 s, annealing at 60 °C for 30 s and elongation at 72 °C for 30 s, with a final extension step at 72 °C for 10 min. The obtained amplicons were labeled with biotin-16-dUTP and digoxigenin-11-dUTP by PCR according to the manufacturer’s instructions (Roche, Germany). To identify V-genome chromosomes in the wheat introgression lines, the FISH probe Dv1, based on a specific *Gypsy*-like retrotransposon sequence [52], was used, while the standard oligo-probes oligo-pAs1-1, oligo-pSc119.2-2 and GAA_n_ [53,54] were used to identify bread wheat chromosomes. Localization of the repeats on the chromosomes of the introgression lines was carried out by fluorescence in situ hybridization (FISH) according to the protocol of [55]. For detection, streptavidin-Cy3 (Vector Laboratories, Peterborough, UK) and anti-digoxigenin-FITC (Roche, Mannheim, Germany) were used. After hybridization, chromosomes were counterstained with DAPI in Vectashield mounting medium (Vector Laboratories, Peterborough, UK). Signals were captured using a DFC 9000 GTC fluorescence microscope (Leica, Wetzlar, Germany) and processed in Adobe Photoshop 2017.1.1 (Adobe Inc., San Jose, CA, USA). At least 10 metaphase plates per preparation were analyzed.

## 5. Conclusions

Comparative analysis of satellite repeat copy-number profiles revealed distinct patterns associated with the genomic and taxonomic structure of the studied Triticeae species. Based on Spearman correlation analysis, E7 and E9 were identified as putative candidates statistically associated with St/StY genomic backgrounds, whereas E10 showed a significant association with the H genomic component. These associations should be considered preliminary and require independent cytogenetic validation across a broader taxonomic panel before subgenome specificity can be established.

Repeats E6 and E11 were experimentally validated as V-chromosome-associated markers in *Triticum aestivum*–*Dasypyrum villosum* introgression lines. Combined qPCR and FISH analyses confirmed their differential distribution among V chromosomes and also clarified the karyotypic status of the W3, W4, and W7 lines.

The repeatome and chromosome-number data also revealed atypical accession-level patterns in *E. dahuricus* PI 634270 and *E. barbicallus* PI 504441. Because each species was represented by a single accession, these observations should not be generalized to the species level. Their precise genomic composition remains unresolved and requires further cytogenomic characterization. Overall, E6 and E11 represent experimentally validated V-chromosome-associated markers, whereas E7, E9, and E10 constitute statistically supported candidates for further cytogenetic validation and comparative genomic studies within the tribe Triticeae.

## Figures and Tables

**Table 1:** Relative copy number (log_10_) of clusters E6 and E11 in *T. aestivum* (Chinese Spring), *D. villosum*, and disomic *T. aestivum*–*Dasypyrum* addition lines.

Line	Added *D. villosum* Chromosome	E6, log_10_(RQ)	E11, log_10_(RQ)
*T. aestivum* (Chinese Spring)	– (control)	1.31	−2.50
*D. villosum* W6 21717	– (control, all V chromosomes)	4.98	4.97
W1	1V	1.14	−2.12
W3	2V	2.22	−2.07
W4	4V	2.39	3.54
W5	5V	1.20	−0.72
W6	6V	1.39	0.50
W7	7V	2.74	−0.78

**Table 2:** Plant material used in the study, includes species and accession, genomic formula, chromosome number (2n), experimental application (qPCR and/or sequencing), origin and source of plant material.

Species	Accession	2n	Genome Formula	Purpose (qPCR/Sequencing)	Origin	Source
*E. tschimganicus*	PI 14438	42	StHY	qPCR, sequencing	Kazakhstan	GRIN ^1^
*E. tsukushiensis*	PI 283170	42	StHY	qPCR, sequencing	Japan	GRIN
*E. dahuricus*	PI 634270	28	-*	qPCR, sequencing	Russia	GRIN
*E. barbicallus*	PI 504441	35	-	qPCR, sequencing	China	GRIN
*E. pendulinus*	PI 639804	28	StY	sequencing	Mongolia	GRIN
*E. arizonicus*	PI 531558	28	StH	sequencing	United States	GRIN
*E. caninus*	-	42	StStH	qPCR, sequencing	Russia	FWRC FPA ^2^
*E. repens^(Afg)^*	PI 223234	42	StStH	qPCR, sequencing	Afganistan	GRIN
*E. repens^(Rus)^*	-	42	StStH	qPCR, sequencing	Russia	FWRC FPA
*P. spicata*	PI 578855	14	St	qPCR	New Mexico, United States	GRIN
*P. tauri*	PI 380652	14	St	qPCR	Iran	GRIN
*D. villosum*	W6 21717	14	V	qPCR	Ukraine	GRIN
*D. breviaristatum*	PI 516547	28	VV^b^	qPCR, sequencing	Morocco	GRIN
*T. aestivum*	Chinese Spring	42	BAD	qPCR	-	-
*T. durum*	Yagut	28	AB	qPCR	-	-
*S. cereale*	Bereginya	14	R	qPCR	-	-
*H. vulgare*	Jilin	14	H	qPCR	-	FRC «Nemchinovka» ^3^

GRIN ^1^—USDA- ARS Germplasm Resources Information Network (Agricultural Research Service, United States Department of Agriculture). FWRC FPA ^2^— Federal Williams Research Center of Forage Production & Agroecology. FRC «Nemchinovka» ^3^— Federal Research Center «Nemchinovka». *—Several specimens displayed an atypical chromosome number, precluding the direct assignment of the standard genomic formula (it is reported that *E. dahuricus* 2n = 42 has the genomic formula StHY [45], *E. barbicallus* 2n = 28—StY [41]) to these particular accessions.

**Table 3:** Repeats and primers used for probe amplification and qPCR analysis.

Cluster	Origin	Primers
E1	*E. tsukushiensis*	F: 5′-CCTTTGACTTTCGCCGGAC-3′ R: 5′-CGACACGGAGGGAATCTTGC-3′
	*E. tschimganicus*	
	*E. dahuricus*	
	*E. caninus*	
	*E. repens^(Afg)^*	
	*E. repens^(Rus)^*	
E2	*E. tsukushiensis*	F: 5′-GTGCGTTTACGTGTCGGTCA-3′ R: 5′-AGTAATAGTCCACGAAACGGGC-3′
	*E. tschimganicus*	
	*E. dahuricus*	
	*E. caninus*	
	*E. repens^(Afg)^*	
	*E. repens^(Rus)^*	
E3	*E. tsukushiensis*	F: 5′-CGATTCAGTAGGAAGCGGGT-3′ R: 5′-AAAATGCGGTCAAAACGGCG-3′
	*E. tschimganicus*	
	*E. dahuricus*	
	*E. caninus*	
E4	*E. tsukushiensis*	F: 5′-TCGTCCGAAACCCTGATACT-3′ R: 5′-AGGGTTACGGCAAAAACTGGA-3′
	*E. tschimganicus*	
	*E. caninus*	
	*E. repens^(Afg)^*	
	*E. repens^(Rus)^*	
E5	*E. tschimganicus*	F: 5′-TTGGATGGCCACTGACCAAG-3′ R: 5′-TGGCAATTTTCAGGACCAAACT-3′
	*E. dahuricus*	
	*E. caninus*	
E6	*E. tsukushiensis*	F: 5′-ACTACCTTTTCAAGCCACCGT-3′ R: 5′-GGAGGTCATATATGGAGACCTATTT-3′
	*E. tschimganicus*	
	*E. dahuricus*	
	*E. caninus*	
	*E. repens^(Afg)^*	
	*E. repens^(Rus)^*	
E7	*E. tsukushiensis*	F: 5′-CACATGGGATGCCAACTGC-3′ R: 5′-TGGTCGAAACTAGAGCACACT-3′
	*E. tschimganicus*	
	*E. dahuricus*	
	*E. caninus*	
	*E. repens^(Afg)^*	
	*E. repens^(Rus)^*	
E8	*E. repens^(Afg)^*	F: 5′-CACGCAAGAGTTGAGCGAAA-3′ R: 5′-GACGCTCGGTGCATTTCCTA-3′
	*E. tsukushiensis*	
E9	*E. tsukushiensis*	F: 5′-GCACATGGTGTACGTGATGG-3′ R: 5′-TTGATTTCCGACGTTCGATGC-3′
E10	*E. repens^(Afg)^*	F: 5′-CCTTCGTCCTTTGCCCTTGA-3′ R: 5′-AGCACGAGCATGGTTTTCCA-3′
	*E. tsukushiensis*	
E11	*E. tsukushiensis*	F: 5′-GTAGACGCCCCACCAATGA-3′ R: 5′-GCTTTCCAACGCCACTGAAA-3′
E12	*E. repens^(Afg)^*	F: 5′-ACTCACTGATTTTGGGTCCCG-3′ R: 5′-TCTGCGAGTTTTGGCGAGG-3′
	*E. tsukushiensis*	
E13	*E. repens^(Afg)^*	F: 5′-AGGAGTGGCAAGAGCCTAAG-3′ R: 5′-GTAGACGAAAGAGGGGATGC-3′
	*E. tsukushiensis*	
E14	*E. repens^(Afg)^*	F: 5′-ATGCTCTATCACCCATCCCG-3′ R: 5′-CAAGGTAAGTTGATCGCGCC-3′
	*E. tsukushiensis*	
E15	*E. barbicallus*	F: 5′-AGCGCATTGCATCCATCTTG-3′ R: 5′-CTCGTCCGGTCTATGATTCGG-3′
E16	*E. barbicallus*	F: 5′-TAACGGGCAAGCTATGGAGC-3′ R: 5′-AAGCGGCTACAAAGAGGGAC-3′

## Data Availability

The original contributions presented in this study are included in the article/Supplementary Material. Further inquiries can be directed to the corresponding author.

## Supplementary Materials

The following supporting information can be downloaded at: https://www.mdpi.com/article/10.3390/ijms27167362/s1.
